# The complete chloroplast genome sequence of medicinal plant, *Selaginella involvens*

**DOI:** 10.1080/23802359.2020.1781574

**Published:** 2020-06-26

**Authors:** Dangying Qiu, Yangmin Wen, Yonghua Xie, Qingfan Lin

**Affiliations:** Quanzhou Medical College, Quanzhou, China

**Keywords:** *Selaginella involvens*, chloroplast genome, phylogenetic analysis, genetic information

## Abstract

*Selaginella involvens* distributed in East Asia region including China used as traditional medicine, which is an important medicinal plant for preventing and treating asthma. The complete chloroplast genome sequence of *S. involvens* was characterized from Illumina pair-end sequencing. The chloroplast genome of *S. involvens* was 126,340 bp in length, containing a large single-copy region (LSC) of 53,214 bp, a small single-copy region (SSC) of 47,561 bp, and two inverted repeat (IR) regions of 12,796 bp. The overall GC content is 38.70%, whereas the corresponding values of the LSC, SSC, and IR regions are 36.2%, 31.9%, and 43.2%, respectively. The genome contains 80 complete genes, including 61 protein-coding genes (45 protein-coding gene species), nine tRNA genes (six tRNA species), and eight rRNA genes (four rRNA species). The Neighbour-joining phylogenetic analysis showed that *S. involvens* and *Selaginella tamariscina* clustered together as sisters to other *Salvia* species.

## Introduction

*Selaginella involvens* distributed in East Asia region including China used as traditional medicine, which is an important medicinal plant for preventing and treating asthma, which has persisted largely in an undomesticated state that is highly resistant to different environmental stresses. Asthma refers to a disease caused by environmental and genetic factors, with dementia as the main clinical phase. Modern pharmacological studies have shown that *S. involvens* can significantly increase the number of capillary networks and accelerate blood flow, thereby restoring the function of microcirculation. At the same time, it can also reduce plasma lactic acid content and improve metabolic disorders caused by cell hypoxia. It plays an important role in preventing and treating asthma. Studies have shown that *S. involvens* can reduce the content of lipid peroxide and erythrocyte sorbitol, increase the level of superoxide dismutase, reduce the oxidative stress reaction, and has an antioxidant effect. Studies have also found that *S. involvens* can improve the role of asthma. The mechanism may be the antioxidant effect of drugs, by restoring the structure and function of nerve cells, inhibiting lipid peroxidation, improving blood rheology, and protecting against ischemia. Damaged neurons improve the pathological response of asthma. Since it contains selaginellin, flavonoids, and sumaflavone, it also presented anti-cancer, inhibition of immediate allergic reactions, and antihyperglycemic activities. *S. involvens* has high ecological and economic value with high levels of intraspecific genetic diversity. *S. involvens* has wide geographic distribution, high intraspecific polymorphism, adaptability to different environments, combined with a relatively small genome size. Consequently, *S. involvens* represents an excellent model for understanding how different evolutionary forces have sculpted the variation patterns in the genome during the process of population differentiation and ecological speciation (Neale and Antoine 2011). Moreover, we can develop conservation strategies easily when we understand the genetic information of *S. involvens*. In the present research, we constructed the whole chloroplast genome of *S. involvens* and understood many genome variation information about the species, which will provide beneficial help for population genetics studies of *S. involvens.*

The fresh leaves of *S. involvens* were collected from Yanzhou (116°35′E; 35°43′N). Fresh leaves were silica-dried and taken to the laboratory until DNA extraction. The voucher specimen (YZJB001) was laid in the Herbarium of Quanzhou medical college and the extracted DNA was stored in the −80 °C refrigerator of the Key Laboratory of Quanzhou Medical College. We extracted total genomic DNA from 25 mg silica-gel-dried leaf using a modified CTAB method (Doyle 1987). The whole-genome sequencing was then conducted by Biodata Biotechnologies Inc. (Hefei, China) with Illumina Hiseq platform. The Illumina HiSeq 2000 platform (Illumina,San Diego, CA) was used to perform the genome sequence. We used the software MITObim 1.8 (Hahn et al. 2013) and metaSPAdes (Nurk et al. 2017) to assemble chloroplast genomes. We used *Selaginella tamariscina* (GenBank: NC041646) as a reference genome. We annotated the chloroplast genome with the software DOGMA (Wyman et al. 2004), and then corrected the results using Geneious 8.0.2 (Campos et al. 2016) and Sequin 15.50 (http://www.ncbi.nlm.nih.gov/Sequin/).

The complete chloroplast genome of *S. involvens* (GenBank accession number MT472526) was characterized from Illumina pair-end sequencing. The complete chloroplast genome sequence of *S. involvens* was characterized from Illumina pair-end sequencing. The chloroplast genome of *S. involvens* was 126,340 bp in length, containing a large single-copy region (LSC) of 53,214 bp, a small single-copy region (SSC) of 47,561 bp, and two inverted repeat (IR) regions of 12,796 bp. The overall GC content is 38.70%, whereas the corresponding values of the LSC, SSC, and IR regions are 36.2%, 31.9%, and 43.2%, respectively. The genome contains 80 complete genes, including 61 protein-coding genes (45 protein-coding gene species), nine tRNA genes (six tRNA species), and eight rRNA genes (four rRNA species).

**Figure 1.**
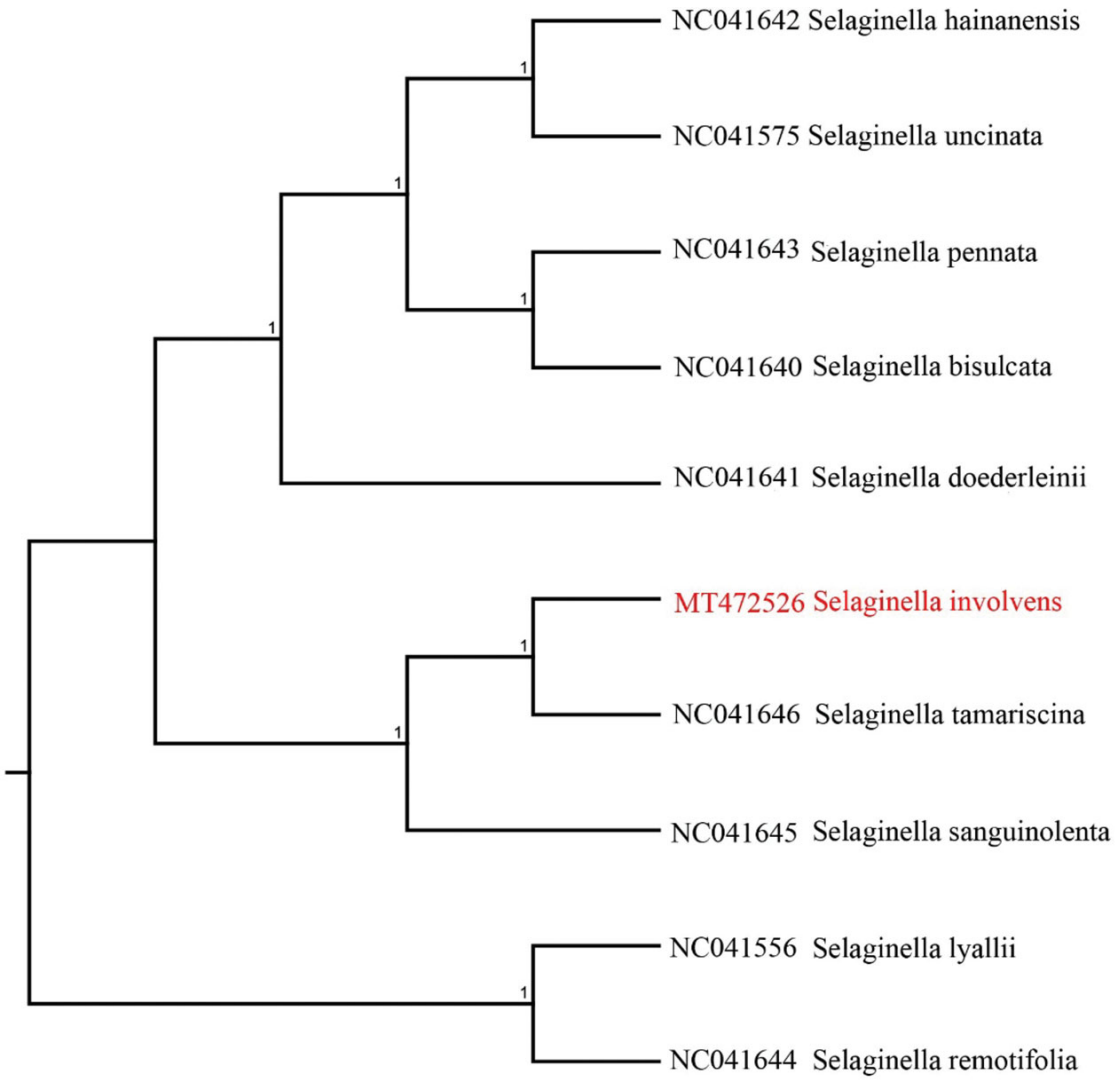
Neighbour-joining (NJ) analysis of *Selaginella involvens* and other related species based on the complete chloroplast genome sequence.

We used the complete chloroplast genomes sequence of *S. involvens* and nine other related species to construct phylogenetic tree. The 10 chloroplast genome sequences were aligned with MAFFT (Katoh and Standley 2013), and then the Neighbour-joining tree was constructed by MEGA 7.0 (Kumar et al. 2016). The Neighbour-joining phylogenetic analysis showed that *S. involvens* and *Selaginella tamariscina* clustered together as sisters to other *Salvia* species (Figure 1).

## Data Availability

The data that support the findings of this study are openly available in GenBank at https://www.ncbi.nlm.nih.gov, reference number MT472526.
